# Escaping endogenous miRNA post-transcriptional silencing of *JrGRF4b* enhanced transformation efficiency in woody plants

**DOI:** 10.3389/fpls.2025.1629166

**Published:** 2025-06-18

**Authors:** Baoxin Li, Mengting Pan, Dezheng Wu, Zhimin Zheng, Dong Pei

**Affiliations:** ^1^ State Key Laboratory of Tree Genetics and Breeding, Research Institute of Forestry, Chinese Academy of Forestry, Beijing, China; ^2^ State Key Laboratory of Tree Genetics and Breeding, College of Forestry, Northeast Forestry University, Harbin, China

**Keywords:** walnut, birch, MiR396, growth-regulating factors, post-transcriptional regulatory, transgene

## Abstract

The stable expression of transgenes was critically influenced by post-transcriptional regulatory mechanisms in transgenic plants. In this study, we investigated the influence of endogenous miRNA-mediated silencing on heterologous gene expression by introducing walnut (*Juglans regia* L.)-derived Growth-Regulating Factors 4 (*JrGRF4b*), disrupting miR396-mediated silencing of replace-*JrGRF4b* (*rJrGRF4b*), and *Jr-miR396a* into birch (*Betula platyphylla* Suk.). While *JrGRF4b* overexpression showed no significant improvement in transformation efficiency due to *Bp-miR396*-mediated suppression, transgenic lines expressing *rJrGRF4b* exhibited a 2.53% increase in transformation efficiency, along with significantly enhanced callus diameter, adventitious bud height, root elongation, cellular expansion, and shoot primordia proliferation compared to control (**p<0.01). In contrast, *Jr-miR396a*-overexpressing plants displayed growth inhibition through suppression of endogenous *BpGRFs*. The results showed that escaping endogenous miRNA regulation by targeted site modification of *rJrGRF4b* significantly improved transgene performance in woody plants. Thus, comprehensive evaluation of post-transcriptional epigenetic regulation between transgenes and endogenous miRNAs in recipient plants was demonstrated to be important, and targeted escape from such miRNA-mediated suppression was shown to ensure stable and high-efficiency transgene expression.

## Introduction

The genetic transformation of plants was recognized for its potential to address major socioeconomic challenges by providing solutions for essential needs such as food, fiber, fuel, and other resources through plant enhancement (Cheng et al., 2024b; Noack et al., 2024). Therefore, ensuring the stable expression and functional performance of transgenes in recipient plants was considered a critical focus of current research in plant biotechnology (Streatfield, 2007). Plant genetic transformation was first achieved in model species such as *Arabidopsis thaliana* (Lloyd et al., 1986; Bechtold and Bouchez, 1995) and *Nicotiana benthamiana* (Fraley et al., 1983; Herrera-Estrella et al., 1983), and was subsequently extended to crops like *Oryza sativa* L (Zhang et al., 1988; Hiei et al., 1994), *Triticum aestivum* L (Vasil et al., 1992), and *Glycine max* (L.) Merr (Hinchee et al., 1988), as well as to perennial woody species such as *Populus* L (Han et al., 2000), *Betula platyphylla* Suk (Valjakka et al., 2000; Cheng et al., 2024a), and *Citrus reticulata Blanco* (Sugimura et al., 2005). However, many plants, particularly perennial trees, exhibited challenges including the lack of efficient regeneration systems and low transformation efficiencies (Wang et al., 2025). To investigate the functions of key genes, heterologous transformation in alternative species was often required (Heyman et al., 2016). Therefore, the stable expression of the targeted gene in transformed plants was of critical importance for both the study and application of transgenic plants (Cao et al., 2023).After the integration of exogenous gene into the target plant genome, its stable expression was closely related to external environmental factors and internal plant regulatory systems.

On one hand, the environment was changed, such as temperature, light, and the application of plant growth regulators, can alter gene expression (Chaudhry and Sidhu, 2022; Wu et al., 2022; Sato et al., 2024). On the other hand, studies have shown that once exogenous genes are integrated into the plant genome, they are easily suppressed by epigenetic regulatory mechanisms, primarily including DNA methylation and small RNA (sRNA)-mediated silencing (Kan et al., 2022; Zhang and Zhu, 2025). Especially, miRNA played a crucial role in post-transcriptional repression (Betti et al., 2021). Studies revealed that the introduction of exogenous genes often led to the suppression of corresponding endogenous gene expression, co-suppression, which was associated with miRNA-mediated specific post-transcriptional regulatory mechanisms (Chen et al., 2024). Initially, in *Caenorhabditis elegans*, the 22-nt small RNA *lin-4* was discovered to inhibit translation by binding to the 3′-UTR of *lin-14* mRNA (Lee et al., 1993). Subsequently, evolutionarily conserved and diverse miRNAs were found widely across eukaryotes, including drosophila, mammals, and plants (Chen and Kim, 2024). On one hand, miRNAs suppressed translation initiation by targeting the 5′-UTR or 3′-UTR regions, thereby blocking ribosome recruitment (Agarwal et al., 2015; Bartel, 2018). In *Arabidopsis thaliana*, for instance, the miR172 binding sites of several AP2 family members (such as TOE1/TOE2/TOE3, SMZ, and SNZ) were located in their 3′-UTR regions (Karlova et al., 2013). In addition, degradome sequencing identified genes whose 5′-UTR regions also served as miRNA target sites (Gu et al., 2014). On the other hand, miRNAs could induce mRNA degradation through complementary base pairing with transcripts, such as miR156 regulating shoot apical meristem development and tillering in rice via *SPL* genes (Chuck et al., 2010); miR167 targeting *ARF6/8* to control anther development; miR319 modulating *TCPs* factors to affect leaf and petal morphology (Wu et al., 2006); miR396 regulating *GRFs* genes to influence cell proliferation and plant morphogenesis (Rodriguez et al., 2010); and miR857 targeting *LACCASE* genes to regulate secondary growth in plants (Zhao et al., 2015). Growth-Regulating Factors (GRFs) interact with their co-factors, GRF-Interacting Factors (GIFs), which in turn recruited the ATP-dependent DNA translocase Switch/Sucrose Non-fermenting (SWI/SNF) complex to promote chromatin remodeling (Kim, 2019; Liu et al., 2023). This mechanism regulates essential biological processes, including organogenesis, flowering, photosynthesis, and tissue regeneration (Zhang et al., 2018; Chen et al., 2019; Lu et al., 2020). Within this regulatory network, miR396 was identified as an evolutionarily conserved negative regulator that controlled GRF transcript abundance by targeting complementary sequences to trigger mRNA degradation (Baucher et al., 2013; Liu et al., 2014). It has been demonstrated that the GRF4-GIF1 complex protein enhances transformation efficiencies in various annual crops, including wheat, rice, sorghum and watermelon (Debernardi et al., 2020; Feng et al., 2021; Li et al., 2024, 2025). These results suggest that the GRF-GIF strategy is a conserved and effective method for improving transformation across different plant species. In addition, a naturally occurring 2-bp substitution within OsGRF4 was found to disrupt miR396-mediated regulation in rice, leading to increased grain size and enhanced yield (Gao et al., 2015). overexpression of a miR396-resistant form of OsGRF4 was shown to positively influence grain size and yield-related traits in both rice and wheat (Li et al., 2018). Furthermore, a 6-bp in-frame deletion in the miR396 target site of *OsGRF4* by CRISPR-Cas9 technology, resulted in significant increases in grain weight and grain sizes in rice (Wang et al., 2022). The cytosine base of the miR396 binding site was edited by the cytosine base editor A3A-PBE in TaGRF4, which disrupted miR396-mediated posttranscriptional regulation and enlarged bread wheat grain size (Li et al., 2025). Therefore, the expression of *GRFs* genes was modulated by manipulating the miR396 binding site within *GRFs* mRNA, thereby influencing the genetic transformation efficiency, seed size, and yield of transgenic plants. Similarly, due to the highly conserved of the miR396-*GRFs* regulatory relationship across eukaryotes, interactions between the target gene and endogenous related genes within the transgenic recipient plants needed to be carefully considered during functional studies, in order to ensure efficient and stable expression of the introduced genes avoid post-transcriptional silencing.

As a major woody oilseed species, walnut has important scientific and application value for gene function (Ji et al., 2021). However, due to the underdeveloped genetic transformation system in walnut, heterologous transformation has been required for functional research at present. The role of the *Jr-miR396*-*JrGRFs* module in woody plant transformation remained unknown. Therefore, in this study, overexpression vectors of *Jr-miR396a*, *JrGRF4b*, and disrupted a miR396 binding site-disrupted version of *rJrGRF4b* were constructed and integrated into birch, a perennial woody species with a well-established transformation system. *rJrGRF4b* escaped post-transcriptional repression by endogenous *Bp-miR396* in birch, thereby enhanced transformation efficiency and accelerated the transformation process in birch. In contrast, overexpression of *Jr-miR396a* targeted and inhibited endogenous *BpGRFs* genes, resulting in reduced transformation efficiency and delayed transformation cycles. This study established that bypassing endogenous miRNA regulation through target site modification (*rJrGRF4b*) effectively enhanced transgene performance in birch. These findings highlighted the necessity of evaluating potential post-transcriptional epigenetic regulatory interactions between transgenes and endogenous miRNAs in recipient plants, as the results show these assessments are critical for achieving stable and high-level transgene expression during heterologous transformation.

## Materials and methods

### Plant materials and growth conditions

Ten-year-old ‘Qingxiang’ walnut (*Juglans regia* L.) trees were planted in Hebei Province, China. The walnut female flowers and male flowers tissues were collected in April, and leaves, green husks, roots, stems, kernels, and embryos were collected in August for transcriptome sequencing. Matured zygotic embryos were obtained from the superior white birch strain DL-1, cultivated in Harbin with controlled artificial pollination. All zygotic embryos were half-sibling families, and the offspring exhibited stable genetic material with no phenotypic segregation.

### Analysis of transcriptome data

Total RNA extraction was conducted using Easy Fast Plant Tissue KitRNA (TIANGEN, Beijing, China). The integrity and purity of RNA were evaluated through agarose gel electrophoresis and UV spectrophotometric. The library construction was conducted the Novogene NGS RNA Library Pre Kit (Novogene, Tianjin, China). The Agilent 2100 Bioanalyzer and ABI StepOnePlus Real-Time PCR System were employed for quality assessment, with subsequent sequencing performed on the Illumina HiSeq 2500 platform following quality control approval. The clean reads were mapped to the walnut reference genome with HISAT2 software. The RNA-seq data of 8 different walnut tissues can be downloaded from PRJNA721107 (SRR15651918-SRR15651926). Go Annotation of TBtools-II software was used for GO function enrichment analysis. The *JrGRFs* transcript levels in each tissue were derived from FPKM values obtained through transcriptome data analysis.

### Bioinformatics analysis of the JrGRFs family

The AtGRFs, OsGRFs, PtGRFs and BpGRFs protein sequences obtained from plant transcription factor database (https://planttfdb.gao-lab.org/) were used to conduct a BLASTp search of the walnut reference genome. The resultant JrGRFs candidate proteins were evaluated to determine whether they contained a QLQ domain (PF08880) and a WRC domain (PF08879) utilizing the Pfam (http://pfam.xfam.org/) databases. Phylogenetic analyses were conducted by using MEGA X to construct a phylogenetic tree using a maximum likelihood method and full-length GRF protein sequences derived from walnut, *Arabidopsis thaliana*, rice, *Populus trichocarpa* and birch. Branch support was assessed with 1000 bootstrap replicates, and nodes with bootstrap values ≥80% were considered well-supported.

### Prediction of binding energy and detection of off-target effects

The secondary structure of the *Jr-miR396a* precursor was predicted bioinformatically in RNAfold WebServer (http://rna.tbi.univie.ac.at//cgi-bin/RNAWebSuite/RNAfold.cgi) by extending 150 bp upstream and downstream of the mature sequence (Meyers et al., 2008). The binding energy between miR396 and *GRFs* mRNAs were calculated using the IntaRNA online tool, with a mismatch threshold of 0–2 and an RNA bulge size of 0.

### Vector construction

To generate the JrGRF4b overexpression constructs, their DNA sequences were obtained PCR amplification from walnut cDNA. Similarly, the precursor sequence of *Jr-miR396a-1* was isolated from the walnut genome during the vector assembly process. To prevent changes in the encoded amino acid sequence, seven nucleotides within the *JrGRF4b* target regions were precisely altered using site-directed mutagenesis primers, with overlapping PCR produced *rJrGRF4b* sequence (**Supplementary Table S1**). The resulting fragments were inserted into the pEASY-Blunt Zero plasmid separately, sequence-verified through Sanger sequencing, and subsequently integrated into the *pMCP1* plant expression system (contained basta resistance gene).

### *Agrobacterium-*mediated transformation in birch

*Agrobacterium*-mediated birch matured zygotic embryos transformation was conducted based on a modified version of a previously published protocol (Cheng et al., 2024a): The process was as follows: A was washed with flowing water for 48–72 hours and then sterilized using 75% alcohol, 30% hydrogen peroxide, and sterile water in a laminar flow hood. Transformation was carried out using the Agrobacterium strain EHA105, with the Agrobacteria grown to an OD_600_ of 0.6-0.8. Matured zygotic embryos was cut using a scalpel in a laminar flow hood, then infected with *Agrobacterium* and placed in co-cultivation medium for dark incubation for 3 days at 26°C. After 3 days, matured zygotic embryos were transferred to a callus induction medium containing basta and incubated under light for 30 days at 26°C. Once the callus expanded, these were transferred to shoot induction mediums containing basta and incubated under light for 30 days at 26°C. Finally, the shoots were cut and inoculated onto roots induction mediums, where they were cultured for 15 days.

### RNA extraction and qRT-PCR

RNA was extracted using the RNAeasy Kit (Cwbio, Jiangsu, China). cDNA was synthesized with the PrimeScript™ RT reagent Kit (TaKaRa, Beijing, China), and prepared for qRT-PCR analysis. miR396a reverse transcription was synthesized with the miRNAs were extracted by the miRcute Plant miRNA Isolation Kit (TIANGEN, Beijing, China). *Jr-miR396a* reverse transcription by the miRNA 1st Strand cDNA Synthesis Kit (by stem-loop) (Taraka, Beijing, China). Transcriptional levels of genes were quantified normalized using *JrGAPDH* as endogenous control in walnut. While the transcriptional levels of genes were quantified normalized using *BpACTIN* as endogenous control in birch. Transcriptional levels of *Jr-miR396a* were quantified using a stem-loop RT-qPCR method, and normalized using *U6* as endogenous control. To ensure consistency and reliability, three biological replicates were included for each experiment. The quantitative data were analyzed using the 2^-△△Ct^ method (Rao et al., 2013). The related primers were listed in **Supplementary Table S1**.

### Paraffin section preparation cell area measurement and blastemates counts

Based on the size of callus, all was immersed in 70% FAA fixative and stored at 4°C. Following standard paraffin sectioning procedures, the fixed callus tissues were dehydrated, cleared, embedded in paraffin wax, and sectioned using the rotary microtome. After sectioning, the samples were dried, dewaxed, stained with TBO, and sealed for observation. Images were captured with Olympus Imager. A2 microscope cameras. Blastematas were identified by dense cell clusters in cytohistology. The areas of dense cell populations were measured using ImageJ.

### Survival ratio, regeneration ratio, transformation efficiency quantification

Survival ratio (%) = (the number of callus/the number of embryos) ×100%. Regeneration ratio (%) = (the number of callus with shoots/the number of callus) ×100%. Transformation efficiency (%) = (the number of positive shoots/the number of callus with shoots) ×100%.

### Phenotypic analysis

The diameter of the callus was measured by the distance from the center of the callus to both ends, with each callus being measured three times to obtain an average value. For each group, 30 callus samples were measured as biological replicates. Adventitious buds height refers to the height from the base of the lowest adventitious buds to the top of the highest adventitious buds on each adventitious buds. 30 adventitious buds samples were measured as biological replicates. The height of rooted seedlings height refers to the seedlings from the base of the lowest seedlings to the top of the highest seedlings on each seedling. 30 rooted seedlings samples were measured as biological replicates. The rooting rates were determined by the ratio of seedlings with roots to the total number of seedlings after 15 days of culture in rooting induction mediums. For each group, 10 rooting induction mediums samples were measured as biological replicates. There are 10–15 seedlings in each root induction medium. The number of roots refers to the count of adventitious roots emerged from the basal stem segment of each seedling after 15 days of growth in the rooting induction medium. For each group, 30 rooted seedlings samples were measured as biological replicates. The root length refers to the distance from the base of the stem segment to the root tip after 15 days of cultivation in the rooting induction medium. The average root length for each rooted seedling was calculated by averaging the lengths of all its adventitious roots. For each group, 30 rooted seedlings samples were measured as biological replicates.

### Statistical analysis

Statistical analyses were performed with SPSS19. Data. Statistical significance of the data was analyzed using either the Student’s t-tests or Tukey’s multiple range test. Graphs and charts were generated using TBTools II, Origin2021, Microsoft Excel 2016, and Adobe Illustrator 2021.

## Results

### Identification of JrGRFs family and selection of JrGRF4b as the focal point

**Figure 1:**
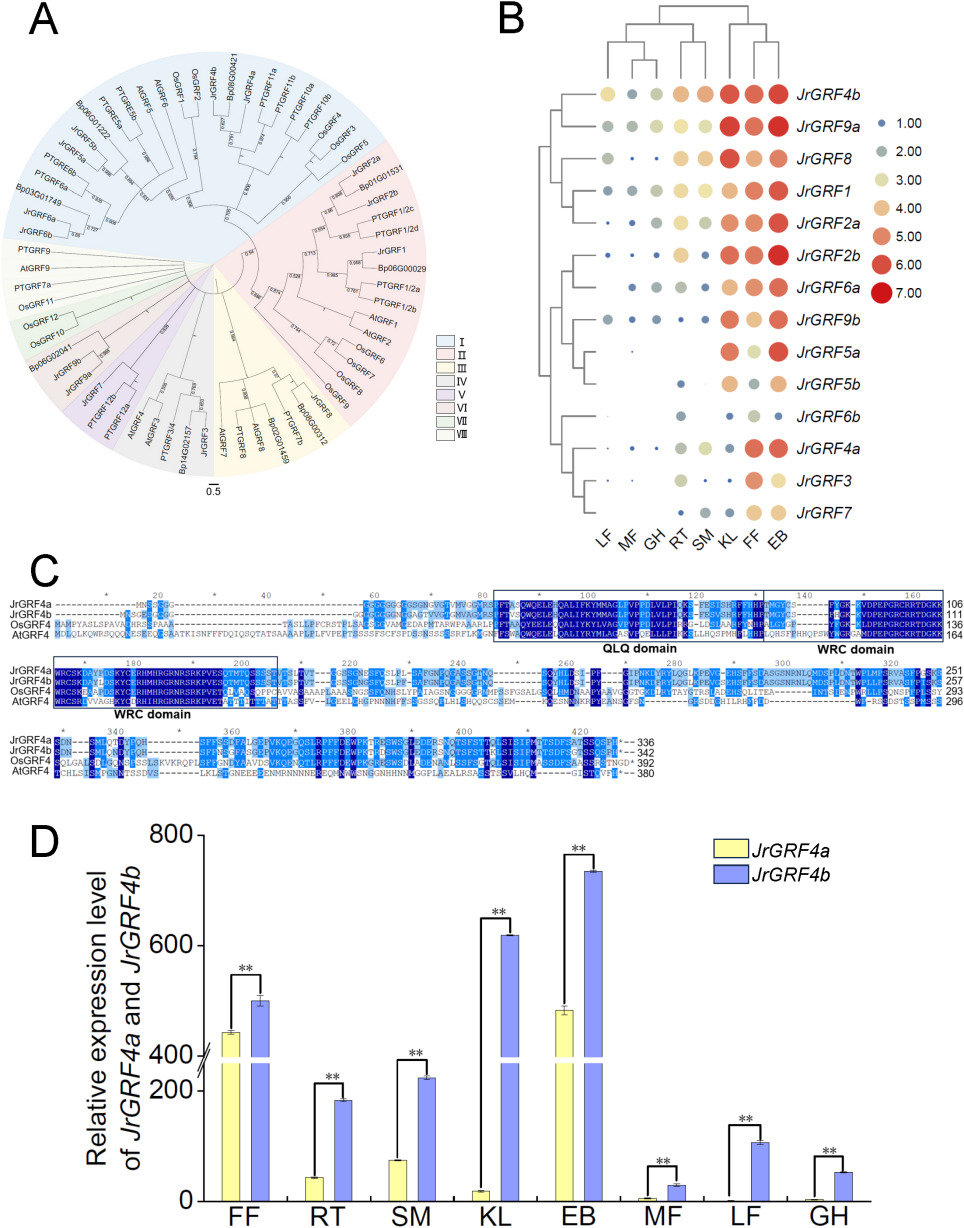
Walnut JrGRFs family classification and expression. **(A)** Phylogenetic analyses of the walnut, *Arabidopsis*, rice, and birch GRFs families. **(B)** Transcript levels of *JrGRFs* genes during the organogenesis of walnut. **(C)** Multiple sequence alignment of JrGRF4a and JrGRF4b with AtGRF4 and OsGRF4 proteins. Conserved QLQ and WRC domains in GRF family proteins were highlighted using black boxes. **(D)** The expression of *JrGRF4a* and *JrGRF4b* during the organogenesis of walnut. LF, leaf; MF, male flower; GH, green husk; RT, root; SM, stem; KL: kernel; FF, female flower; EB, embryo. The error bars represent the SE of three independent biological replicates (t-test: **p < 0.01).

Based on BLASTp (https://blast.ncbi.nlm.nih.gov/) and HMMsearch tools, 14 JrGRFs family members were identified in the walnut genome after filtering the redundant transcripts. We named *JrGRF1*, *JrGRF2a*, *JrGRF2b*, *JrGRF3*, *JrGRF4a*, *JrGRF4b*, *JrGRF5a*, *JrGRF5b*, *JrGRF6a*, *JrGRF6b*, *JrGRF7*, *JrGRF8*, *JrGRF9a*, and *JrGRF9b* basing on protein sequence phylogenetic subfamily classification and chromosome location information. The evolutionary classification of 9 AtGRFs, 12 OsGRFs, 19 PtGRFs, 9 BpGRFs and 14 JrGRFs based on their amino acid sequences showed that these protein members could be divided into eight clusters, including I-VIII. The JrGRFs protein sequences were distributed across clusters I-VI. Cluster I was composed of 6 members, Cluster II consisted of 3 members, Clusters III-V each contained 1 member, and Cluster VI included 2 members. Notably, the JrGRFs of *Juglans regia* L., which were clustered on the same evolutionary clade, were positioned closer to GRFs of *Betula platyphylla* Suk. and *Populus tomentosa* Car., indicating a closer relationship among these families (**Figure 1A**). GO annotation analysis of *JrGRFs* genes revealed mappable pathways exclusively for *JrGRF3*, *JrGRF4b*, *JrGRF5a*, *JrGRF5b*, *JrGRF8*, *JrGRF9a*, and *JrGRF9b*. Subsequent enrichment analysis demonstrated three functional categories: molecular functions (MF), cellular components (CC), and biological processes (BP) (**Supplementary Figure S1**). In the MF category, 11 GO terms were enriched, among which 9 transcription-related terms (such as GO:0003700, GO:0003674, and GO:0003676 etc.) were annotated to *JrGRF3*, while two nucleotide-binding terms (GO:0005524-ATP binding and GO:0005525-GTP binding) were annotated to *JrGRF4b*. In the CC category, 11 GO terms were annotated. Among these, two terms (GO:0005874-microtubule and GO:0005634-nucleus) were exclusively assigned to *JrGRF4b*, while the remaining 10 organelle development-related terms were annotated to other *JrGRFs*. In the BP category, 41 GO terms were annotated. The *JrGRFs* were predominantly enriched in the following plant grow and metabolic pathways (GO:0007275, GO:0008105, GO:0032501, GO:0048366, GO:0048367, GO:0048731, GO:0048827, and GO:0048856). *JrGRF4b* was primarily annotated with terms associated with plant organ development and transcriptional regulation (GO:0099402, GO:0006351, GO:0006355, and GO:0007017). Additionally, *JrGRF8* was found to be associated with light-responsive terms (GO:0009314, GO:0009416, GO:0009628, GO:0009639, GO:0010114, GO:0010218, and GO:0080167) (**Supplementary Figure S1**). These results demonstrated that *JrGRFs* were functionally associated with plant growth and metabolism.

To investigate the involvement of *JrGRFs* genes in the growth and development of walnut trees, RNA-seq expression patterns of 14 *JrGRFs* genes were examined across various walnut tissues. Our study revealed that *JrGRFs* exhibited different expression patterns in eight tissues of walnut (**Figure 1B**). Among them, all 14 *JrGRFs* genes expressed in KL (kernels), FF (female flowers), and EB (embryos). In RT (roots), 13 *JrGRFs* genes (except for *JrGRF5a*) showed detectable expression levels; in SM (stems) and MF (male flowers), 11 *JrGRFs* genes (except for *JrGRF5a*, *JrGRF5b* and *JrGRF6b*) were expressed. In LF (leaves), 10 genes (except for *JrGRF5a*, *JrGRF5b*, *JrGRF6a* and *JrGRF7*) exhibited expression, while only 9 genes (except for *JrGRF3, JrGRF5a*, *JrGRF5b*, *JrGRF6b* and *JrGRF7*) were expressed in GH (green husks). The expression levels of *JrGRFs* genes in KL (kernels), FF (female flowers), and EB (embryos) were higher than in other tissues. In addition, except for MF (male flowers) and GH (green husks), the expression of *JrGRF4b* was higher than that of the other *JrGRFs* genes.

Furthermore, two JrGRFs protein sequences, JrGRF4a and JrGRF4b, were identified in the walnut genome, which conserved QLQ and WRC domains with OsGRF4 in rice and AtGRF4 in *Arabidopsis thaliana*, respectively (**Figure 1C**). Meanwhile, the relative expression analysis of *JrGRF4a* and *JrGRF4b* in eight different walnut tissues revealed that *JrGRF4b* expressed at significantly higher levels than *JrGRF4a* across all tissues by qRT-PCR (**p<0.01, **Figure 1D**). The result indicated that, JrGRF4b might play a more important role in the growth and development of walnut tissues compared to JrGRF4a.

### The characterization and expression of miR396 in walnut

**Figure 2:**
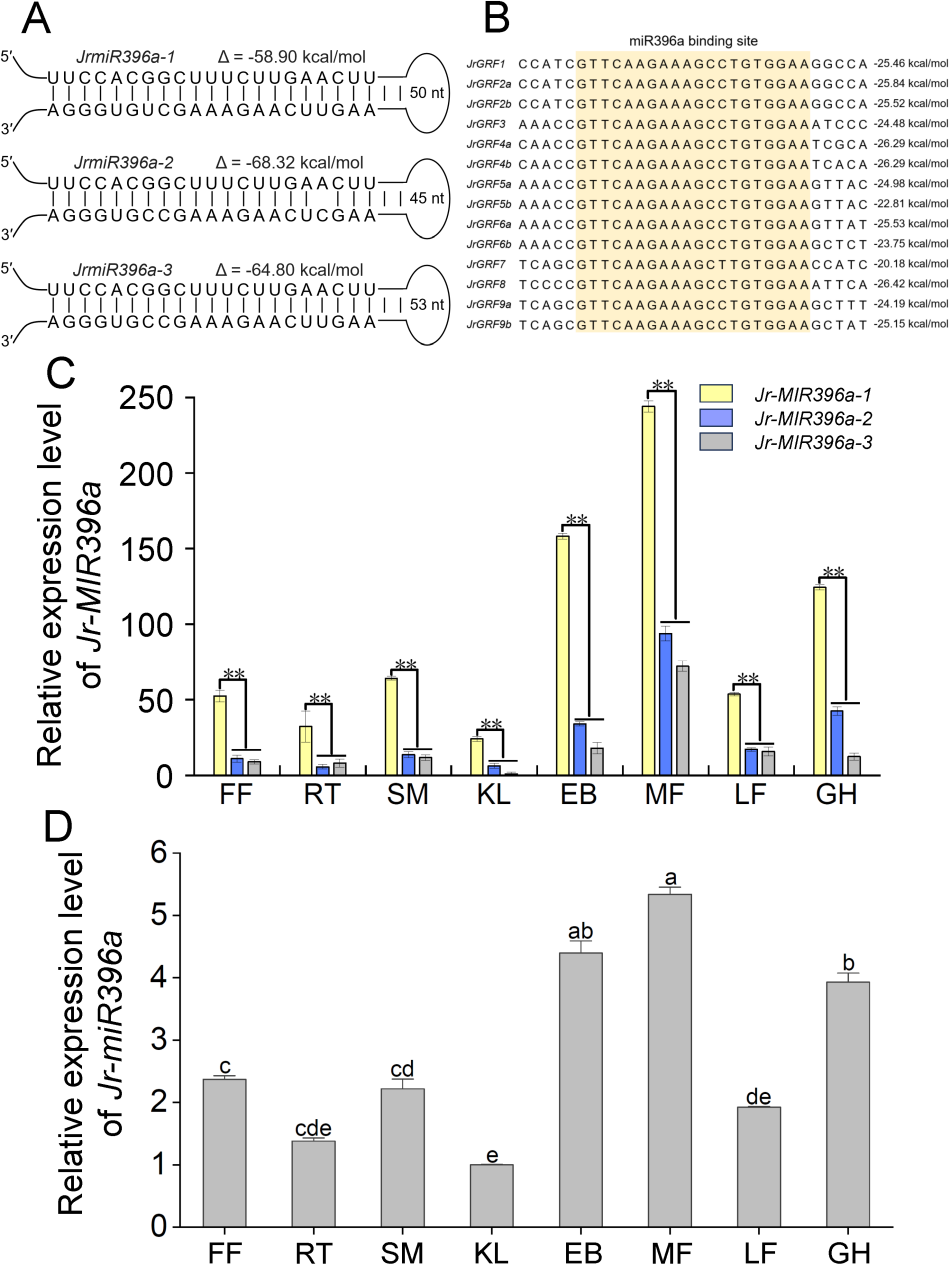
Walnut miR396 identification and expression. **(A)** Secondary structures of miR396 stem-loop precursors. The minimum free energy of the RNA secondary structure was calculated as ΔG (kcal/mol). **(B)** The miR396a binding site of *JrGRFs* genes. The miR396a binding site is marked in orange. The value on the right represents the binding energy of miR396 with *JrGRFS* mRNA. **(C)** The relative expression level of *Jr-MIR396a*. **(D)** The relative expression level of mature *Jr-miR396a*. LF, leaf; MF, male flower; GH, green husk; RT, root; SM, stem; KL: kernel; FF, female flower; EB, embryo. a, b, c, d, and e indicated extremely significant differences among the groups. The error bars represent the SE of three independent biological replicates (t-test: **p < 0.01).

GRFs are regulated by miR396 have been identified in different plant species, including *Arabidopsis thaliana*, rice, and soybean etc (Kim, 2019; Gao et al., 2015; Li et al., 2025). Based on the mature and precursor sequences of miR396 in miRbase, we mapped the *Jr-miR396a-1*, *Jr-miR396a-2* and *Jr-miR396a-3* loci to chromosomes 1, 3 and 6 (**Figure 2A**; **Supplementary Table S1**). The precursor structure of miR396a possessed a complete stem-loop configuration, with the mature sequence of miR396a located in the stem location (**Supplementary Figure S2**). But the mature sequence of miR396a was came from different chromosomes. We further predicted that all 14 *JrGRFs* genes contained the targeted DNA sequence of miR396 conservatively (**Figure 2B**). There were highly conserved binding interactions between miR396 and the *JrGRFs* mRNA, and the binding free energy ranges were from -20.18 kcal/mol to -26.29 kcal/mol (**Supplementary Figure S3**). To further confirmed the expression patterns of the three *Jr-MIR396a*, relative expression levels were analyzed across eight tissues (leaf, male flower, green husk, root, stem, kernel, female flower, and embryo) by qRT-PCR. The results showed that *Jr-MIR396a-1* exhibited significantly higher relative expression than *Jr-MIR396a-2* and *Jr-MIR396a-3* in all examined tissues (**p<0.01). Especially, the relative expression level of *Jr-MIR396a-1* was the highest in ME (male flowers). Meanwhile, we found the relative expression level of *Jr-miR396a* exhibited significantly higher in EB (embryos) and MF (male flowers) than other tissues. Moreover, the expression pattern of mature *Jr-miR396a* in the eight walnut tissues was consistent with that of *Jr-MIR396a*, which suggested that *Jr-MIR396a-1* played a dominant role in this process.

### *rJrGRF4b* accelerated the transformation process of birch

**Figure 3:**
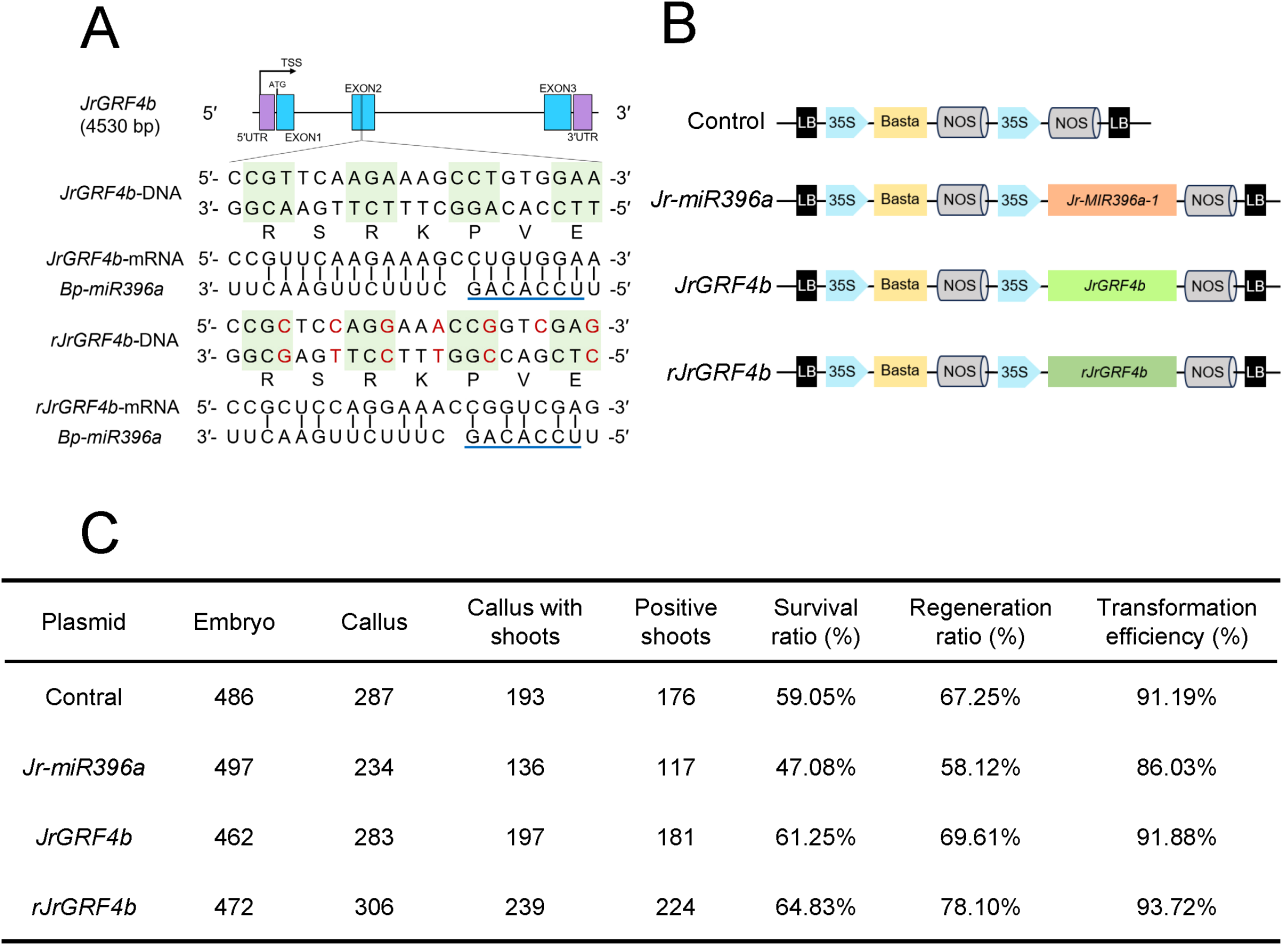
*rJrGRF4b* accelerated the birch transformation process. **(A)** Schematic representation of *JrGRF4b* and *rJrGRF4b* gene structure showing the *BpmiR396a* target site. The *Bp-miR396a*-resistant *rJrGRF4b* version was introduced mutations (in red) to reduce interactions with *Bp-miR396a*. The *Bp-miR396a* seed region (nucleotides 2-8 from 5' to 3') was indicated by the blue highlighted line. **(B)** Schematic construction of the vectors. The expression of *Jr-miR396a*, *JrGRF4b* and *rJrGRF4b* were driven by the *Cauliflowever mosaic virus* 35S promoter (35Sp). The Basta represents the expression cassette for the Basta gene, which serves as a selection marker for transgenic lines. LB and RB, T-DNA left and right borders. **(C)** Overexpression of *rJrGRF4b* increased birch transformation efficiency.

GRF4-GIF1 has been proven to enhance the transformation efficiency of wheat, citrus, sorghum and soybean. To investigate whether *JrGRF4b* could also improve woody plants transformation, such as birch. Firstly, we found that *Bp-miR396a* and *Bp-miR396b* in birch negatively regulated *JrGRF4b* mRNA by targeting it conservatively through bioinformatics prediction (**Supplementary Figure S4**). We created DNA sequence of *rJrGRF4b*, which was disrupted the *Bp-miR396a*, *Bp-miR396b* and *Jr-miR396a* binding sites in mRNA correspondingly (**Figure 3A**; **Supplementary Figures S5**, **S6**). However, the amino acid sequence of rJrGRF4b was same to JrGRF4b, similar to synonymous mutation. Meanwhile, we designed the following vectors: control vector, *Jr-miR396a* vector, *JrGRF4b* vector, and *rJrGRF4b* vector (**Figure 3B**). In all vectors, the expression of *Jr-miR396a*, *JrGRF4b*, and *rJrGRF4b* were driven by the *Cauliflowever mosaic virus* 35S promoter (35Sp), which has been proven to stably drive genes expression in wood plants. Moreover, a 35S-controlled Basta expression module was contained in all vectors to offer glufosinate-ammonium resistance in the transformants (**Figure 3B**).

The genetic transformation process followed the protocol described by matured zygotic embryo of birch infection. In brief, the wounded matured zygotic embryo of birch was infected with *Agrobacterium* suspension at an OD value ranging from 0.6 to 0.8, and then incubated in the dark on co-cultivation medium for 3 days firstly. Subsequently, the surviving callus were transferred to callus induction medium containing glufosinate-ammonium and cultured for 30 days. The selected and enlarged callus were transferred to shoot induction medium and cultured for 30 days until shoots regenerated. Finally, the regenerated shoots were transplanted onto root induction medium and cultured for 15 days until roots regenerated (**Supplementary Figure S7**). We observed both control seedlings and transformed with *Jr-miR396a*, *JrGRF4b*, and *rJrGRF4b* showed no growth defects (**Supplementary Figure S7**). However, there were differences in the growth and development rates among the transgenic plants at various stages of genetic transformation. To further investigated the impact of gene function on genetic transformation, we found that *Jr-miR396a*-OE-1, *JrGRF4b*-OE-7, and *rJrGRF4b*-OE-11 were the highest relative expression levels in callus and seedlings by qRT-PCR respectively (**Supplementary Figure S8**).

**Figure 4:**
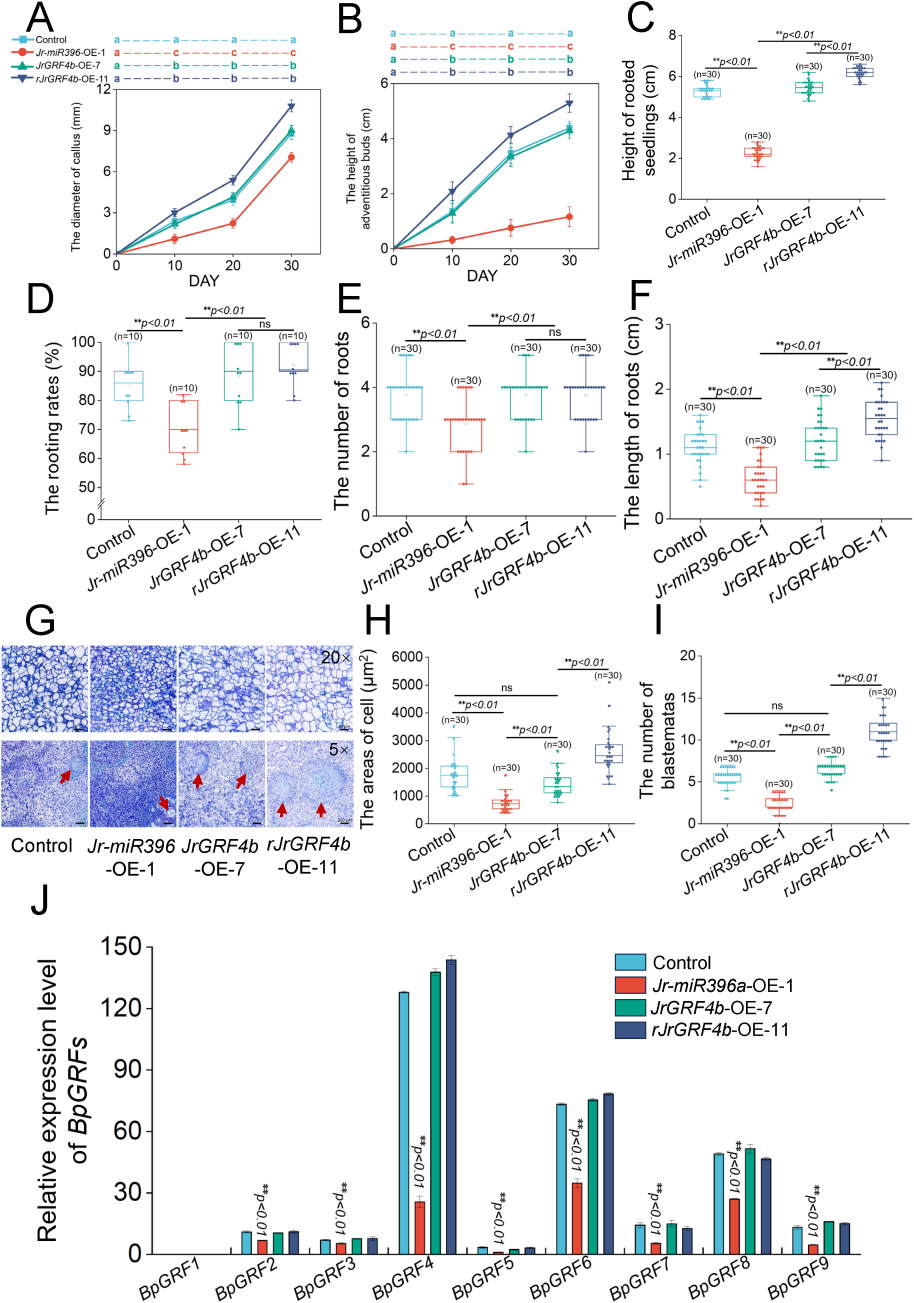
Statistical analysis and morphology of callus cells of transformed with different vectors in birch transformation process. **(A)** The diameter of callus. 30 callus were selected for each period. **(B)** The height of adventitious buds. 30 callus were selected for each period. **(C)** Height of rooted seedings. 30 seedings were selected for each genotype. **(D)** The rooting rates. 30 seedings were selected for each genotype. **(E)** The number of roots. 30 seedings were selected for each genotype. **(F)** The length of roots. 30 seedings were selected for each genotype. **(G)** Histological analysis of callus cells at 30 DAYs. 20× Scale bars = 50 μm, 5×Scale bars = 200 μm. The blastemates were marked by a red arrow. **(H)** The areas of cell. 30 biological replicates were selected for each genotype. **(I)** The number of blastemates. 30 biological replicates were selected for each genotype. **(J)** Relative expression level of *BpGRFs*. The error bars represent the SE of three independent biological replicates (t-test: **p < 0.01).

During the callus induction stage, the callus diameter of *rJrGRF4b*-OE-11 was significantly larger than that of control and *JrGRF4b*-OE-7 (**p<0.01), with no significant difference between control and *JrGRF4b*-OE-7 when cultured on the callus induction medium from 10th day to 30th day of development. In contrast, the callus diameter of *Jr-miR396a*-OE-1 was significantly smaller than the others (**p<0.01, **Figure 4A**). Subsequently, the callus was transferred to regenerated shoots medium, and the cultivation process from 10th day to 30th day of growth, the height of adventitious buds in *rJrGRF4b*-OE-11 was significantly higher than that of control and *JrGRF4b*-OE-7 (**p<0.01), with no significant difference between control and *JrGRF4b*-OE-7. On the contrary, the height of adventitious buds of *Jr-miR396a*-OE-1 was significantly lower than the others (**p<0.01, **Figure 4B**). When adventitious buds were transferred to the rooting medium and cultured for 15th days, the seedlings height of *rJrGRF4b*-OE-11 were higher than those of control and *JrGRF4b*-OE-7 (**p<0.01), with no significant difference between control and *JrGRF4b*-OE-7. However, the height of seedlings of *Jr-miR396a*-OE-1 were significantly lower than the others (**p<0.01, **Figure 4C**). At the same time, there was no significant difference in rooting rates and the number of roots between *JrGRF4b*-OE-7 and *rJrGRF4b*-OE-11, but *Jr-miR396a*-OE-1 was significantly lower than control, *JrGRF4b*-OE-7 and *rJrGRF4b*-OE-11 (**p<0.01, **Figures 4D, E**). However, the roots length of *rJrGRF4b*-OE-11 were longer than those of control and *JrGRF4b*-OE-7 (**p<0.01), and *Jr-miR396a*-OE-1 was significantly lower than control, *JrGRF4b*-OE-7 and *rJrGRF4b*-OE-11 (**p<0.01, **Figure 4F**).

Based on above, whether in callus, adventitious buds, or rooted seedlings, *rJrGRF4b*-OE-11 exhibited a better growth state than control and *JrGRF4b*-OE-7, while the growth and development of transgenic *Jr-miR396a*-OE-1 were clearly inhibited. There was no significant difference in growth condition between control and *JrGRF4b*-OE-7 compared to the same period. This result suggested that *rJrGRF4b* had a potential to accelerate the genetic transformation of woody plants, specifically in birch.

### *rJrGRF4b* improved the transformation efficiency of birch

In order to evaluate the transformation efficiency of the different vectors, 1917 mature zygotic embryos of birch were employed throughout the experiment (**Figure 3C**). For the control vector, 287 out of 486 mature zygotic embryos successfully survived on the callus induction medium, and 197 of callus smoothly regenerated shoots (**Figure 3C**). Furthermore, PCR analysis was performed using specific primers on the regenerated seedlings and we checked to prove that 176 of 193 shoots were transgenic, resulted in the transformation efficiency (positive shoots/callus with shoots) was 91.19% (**Figure 3C**). Among them, JrGRF4b improved the transformation efficiency to 91.88%, rJrGRF4b to 93.72%, while *Jr-miR396a* reduced the transformation efficiency to 86.03%. The above data demonstrated that rJrGRF4b outperformed both the control and JrGRF4b in enhancing the transformation efficiency of birch. Whereas *Jr-miR396a* had the opposite effect (**Figure 3C**).

To investigate the differentiation capacity of control vector, *Jr-miR396a* vector, *JrGRF4b* vector, and *rJrGRF4b* vector during birch transformation, the transformation procedure was further divided into two parts: callus induction and regeneration. Of the 472 mature zygotic embryos transformed with the *rJrGRF4b* vector, 306 formed callus, and 239 of these callus ultimately produced shoots. This led to a callus survival rate of 64.83% and a regeneration efficiency of 78.10%. At the same time, a total of 462 mature zygotic embryos were transformed with the *JrGRF4b* vector, resulting in the formation of callus in 283 cases. Among these, 197 callus successfully regenerated shoots, corresponding to a 64.83% callus survival rate and a 78.10% shoot regeneration efficiency. Both the callus survival ratio and regeneration efficiency of *rJrGRF4b* vector was higher than control vector (59.05% of callus survival ratio and 67.25% of regeneration efficiency), especially in comparison to *JrGRF4b* vector. On the contrary, The *Jr-miR396a* vector was introduced into a total of 497 mature zygotic embryos, leading to callus formation in 234 instances. Of these, shoot regeneration was achieved in 136 callus, a callus survival rate of 47.08% and a regeneration efficiency of 58.12%. The callus survival rate and regeneration ratio were clearly lower than those of control vector. In conclusion, *rJrGRF4b* improved callus survival ratio and shoots regeneration ratio of the transformation efficiency of birch potentially.

### *Jr-miR396a* and *rJrGRF4b* mediated cell development and influenced genetic transformation efficiency in birch

To further elucidate the effects of *Jr-miR396a*, *JrGRF4b*, and *rJrGRF4b* on callus induction and plant regeneration during the genetic transformation process in birch, histological observations were conducted on callus grown for 30th days on callus induction medium. Therefore, we examined the paraffin sections of callus from control, *Jr-miR396a*-OE-1, *JrGRF4b*-OE-7, and *rJrGRF4b*-OE-11 under the light microscope (**Figure 4G**). Under the 20x magnification, on one hand, it was observed that the cell areas of *rJrGRF4b*-OE-11 was significantly larger than that of control and *JrGRF4b*-OE-7 (**p<0.01), with no significant difference between control and *JrGRF4b*-OE-7 (**Figure 4H**). On the other hand, the cell areas of *Jr-miR396a*-OE-1 was significantly smaller than that of control (**p<0.01; **Figure 4H**). Besides, under the 5x magnification, it exhibited a markedly greater number of blastemates compared to both control and *JrGRF4b*-OE-7 (**p<0.01), while control and *JrGRF4b*-OE-7 showed comparable levels without significant difference (**Figure 4I**). As expected, the blastemates numbers was significantly less than control (**p<0.01, **Figure 4I**). In summary, *rJrGRF4b*-OE-11 significantly enhanced callus induction and plant regeneration by promoting the enlargement of callus cells and increasing the number of shoot primordia, whereas *Jr-miR396a*-OE-1 inhibited these processes.

To further investigate the mechanism by which *Jr-miR396a* suppressed genetic transformation efficiency in birch, we proposed the hypothesis that *Jr-miR396a* might target and negatively regulate *BpGRFs* in birch. First of all, we performed bioinformatic predictions for targeted negative regulatory sites of *Jr-miR396a* and *BpGRFs*. The prediction results indicated that all *BpGRFs* genes (*BpGRF1*-*BpGRF9*) were targeted and bound to *Jr-miR396a*, leading to the cleavage of mRNA transcripts and achieving post-transcriptional suppression (**Supplementary Figure S9**). To clarify the potential mechanisms, the relative expression level of *BpGRFs* genes in the callus of control, *Jr-miR396a*-OE-1, *JrGRF4b*-OE-7, and *rJrGRF4b*-OE-11 were analyzed. Except for *BpGRF1*, which was not detected in any of the callus, the relative expression levels of the other seven *BpGRFs* genes (*BpGRF2*-*BpGRF8*) in *Jr-miR396a*-OE-1 were significantly lower than those in control, *JrGRF4b*-OE-7, and *rJrGRF4b*-OE-11 (**p<0.01, **Figure 4J**). The result suggested that overexpression of *Jr-miR396a* down-regulated the expression of *BpGRFs* genes. Therefore, we concluded that *Jr-miR396a* suppressed genetic transformation efficiency in birch most likely by Inhibiting the expression of *BpGRFs* genes to further suppress callus cells growth and differentiation.

## Discussion

### The mechanisms of miR396 were highly conserved in different woody species

Through bioinformatic analyses and homology-based comparisons in the miRNA database, three genomic loci were identified in the walnut reference genome that produced identical mature miR396 sequences, *Jr-miR396a*. Similarly, three loci were detected in the birch reference genome, giving rise to two distinct mature sequences, *Bp-miR396a* and *Bp-miR396b*. However, the seed region sequences (nucleotides 2–8 of from 5′ to 3′, which were important to specific pairing with target mRNA) of the mature miR396 sequences were found to be completely identical between walnut and birch. Furthermore, analysis of the secondary structures of the miR396 precursors revealed that the complementary strand corresponding to the seed region (requirements for biosynthetic mechanisms) at the 3′ regions was also identical between the two species. These results demonstrated that both the mature sequence and precursor secondary structure of miR396 are highly conserved during species evolution in walnut and birch. The biogenesis of miRNAs initiated with the transcription of *MIR* genes in plants by DNA-dependent RNA polymerase II (Pol II), generated primary miRNA transcripts (pri-miRNAs) (Zhan and Meyers, 2023). These single-stranded, polyadenylated pri-miRNAs spontaneously fold into hairpin structures (Borges and Martienssen, 2015). The DCL1 (Dicer-like protein 1) nuclease subsequently cleaved the pri-miRNA to produce precursor miRNAs (pre-miRNAs) containing stem-loop structures (Xie et al., 2004). Further processing by DCL1 yields mature miRNA duplexes, comprising the guide strand (miRNA) and passenger strand (miRNA*) (Zhan and Meyers, 2023). Typically, the miRNA* strand was degraded, while the miRNA strand is incorporated into Argonaute (AGO) proteins to form the RNA-induced silencing complex (RISC). This complex mediates either targeted mRNA cleavage or translational repression, depending on the degree of complementarity between the miRNA and its target sequence (Chen and Kim, 2024). In this study, the *Jr-MIR396a-1* from walnut was successfully integrated birch. Notably, high expression levels of mature *Jr-miR396a* were detected in transgenic plants, demonstrated that endogenous proteins involved in miRNA biogenesis-including BpPol II, BpDCL1, and BpAGOs properly processed the heterologous *Jr-MIR396a-1* into its mature form (included correct hairpin structure formation of *Jr-MIR396a-1*, and accurate cleavage to generate mature *Jr-miR396a* sequences). Furthermore, overexpressing *Jr-MIRR396a-1* of transgenic plants showed significantly reduced expression of endogenous *BpGRFs*, which confirmed that *Jr-miR396a* could assemble into functional RISC complexes with BpAGOs proteins to regulate target genes. In summary, it was demonstrated that the miRNA biosynthetic pathway and its regulatory mechanisms were evolutionarily conserved in both walnut and birch.

### Post-transcriptional silencing of transgenes through miRNA-mediated epigenetic regulation requires careful consideration

There are two distinct modes of miRNA-mediated post-transcriptional repression were identified in plants: (1) translational inhibition through complementary binding to either 5′UTR or 3′UTR regions of target transcripts, and (2) transcript cleavage-induced degradation via perfect base pairing with mRNA targets (Rogers and Chen, 2013). Bioinformatics analysis revealed that *JrGRF4b* mRNA was targeted by *Bp-miR396* for cleavage-mediated suppression in birch. To prevent potential inhibition of walnut-derived *JrGRF4b* by endogenous *Bp-miR396*, a synonymous mutation was introduced into the *JrGRF4b* cDNA sequence, resulting in *rJrGRF4b* (the amino acid sequence was same to *JrGRF4b*) with disrupted *Jr-miR396a* and *Bp-miR396* binding sites. The predictions demonstrated significantly reduced binding affinity of both *Bp-miR396a* and *Bp-miR396b* to *rJrGRF4b* mRNA at two critical regions: the seed sequence (positions 2–8 bp from the 5′ end) and the supplementary binding domain (positions 13–16 bp from the 5′ end), which was previously reported to enhance target recognition (Bartel, 2018). Comparative analysis of transgenic birch lines showed that while overexpression of *JrGRF4b* did not significantly alter transformation efficiency or plant growth characteristics compared to controls, *rJrGRF4b*-overexpressing lines exhibited 2.53% increase in transformation efficiency and significantly improved growth performance at equivalent developmental stages, as evidenced by enhanced callus size, adventitious shoot height, and root length etc.

miRNAs were demonstrated to silence target genes through RISC-mediated mechanisms. AGO proteins, as the primary components of the RISC complex, were characterized by four conserved domains (N, PAZ, MID, and PIWI) (Lee et al., 2023). Among these, the MID domain preferentially selected miRNA guide strands that initiated with uridine (U) or adenine (A) at the 5′ end (Khvorova et al., 2003; Suzuki et al., 2015). Further, the PIWI domain of AGO proteins possessed an RNase H-like fold, and some AGO proteins were capable of cleaving the targeted mRNA between the 10^th^ and 11^th^ positions of the miRNA binding sites (Song et al., 2004). Additionally, during the translational inhibition process, AGO proteins in the RISC complex were found to prevent ribosome assembly or to stall the elongation phase of translation of targeted mRNAs (Bartel, 2018). Therefore, when the target gene was post-transcriptionally repressed by endogenous plant miRNAs, it was important to consider not only the miRNA-target relationships but also to introduce specific synonymous base substitutions based on the functional properties of AGO proteins within the plant. This approach could effectively prevent post-transcriptional silencing.

miRNA-mediated post-transcriptional regulation of target genes was demonstrated to be highly conserved in plants (Chen and Kim, 2024). Beyond the well-characterized miR396-*GRF* module regulating plant growth and development, several other evolutionarily conserved miRNA-target pairs were identified: miR156-*SPL*, which negatively regulated the timing of leaf primordium development and flowering in *Zea mays* (Chuck et al., 2010) and *Arabidopsis* (Wang et al., 2008); miR159-*MYB33*, involved in the negative regulation of anther, pistil and seed development in *Arabidopsis* (Allen et al., 2007); miR164-*CUC*, controlling meristem formation and leaf morphology in *Oryza* (Li et al., 2003); miR169-CCAAT box binding factors (*CBFs*), essential for modulating C gene transcription in *Antirrhinum majus* (Cartolano et al., 2007); and miR393-*TIR1/AFB*, which maintained auxin homeostasis through negative regulation in *Arabidopsis* (Parry et al., 2009). During heterologous genetic transformation, when post-transcriptional silencing occurs due to miRNA-mediated epigenetic regulation, the synonymous mutation-based strategy employed in this study, which disrupts miRNA target sites while maintaining the original amino acid sequence, could be implemented to achieve stable and high-level transgene expression in recipient plants.

### miR396-resistant *GRF* enhances woody plant transformation

The results demonstrated that overexpression of *JrGRF4b* did not significantly alter genetic transformation efficiency in birch, callus size, adventitious height, or rooting percentage compared to controls. In contrast, overexpression of *rJrGRF4b* exhibited significant improvements in all these parameters, indicated its potential to enhance transformation efficiency and shorten the regeneration cycle in woody plants. Conversely, overexpression of *Jr-miR396a* in birch suppressed these traits. Further, the *rJrGRF4b*-overexpressing callus displayed markedly enlarged cells and a higher number of differentiated shoot primordia compared to *JrGRF4b* and control lines, whereas *Jr-miR396a* overexpressing callus showed pronounced growth inhibition in cytological observations. *JrGRF4b* was annotated with molecular function terms related to stimulus response with GO analysis, suggesting its potential role in regulating secondary metabolite biosynthesis during plant growth and development. Previous studies reported that overexpression of *AtGRF2/AtGRF3* increased leaf size by upregulating cell cycle genes, accelerating proliferation and cell expansion in *Arabidopsis* (Kim et al., 2003). *Zm-rGRF1* overexpression enhanced leaf length by increasing cell number, while *ZmGRF10* restricted proliferation, reducing plant height and leaf size in maize (Wu et al., 2014; Nelissen et al., 2015). miR396-mediated suppression of *MsGRFs* activity inhibited root apical meristem growth and cell proliferation in alfalfa (*Medicago sativa*) (Bazin et al., 2013). Collectively, the study suggested that *JrGRF4b*, either through miRNA target site disruption or *Bp-miR396* gene editing, represented a viable strategy to overcome intrinsic limitations in woody plant transformation systems by bypassing miRNA-mediated repression of growth-promoting genes.

## Conclusion

In this study, the walnut-derived miR396-GRF module by introducing *Jr-miR396a*, *JrGRF4b*, and its miRNA-resistant variant *rJrGRF4b* into birch. It was found that *JrGRF4b* overexpression was post-transcriptionally suppressed by endogenous *Bp-miR396*, showing no improvement in transformation efficiency. In contrast, *rJrGRF4b* overexpression, which escaped miRNA-mediated repression, increased transformation efficiency by 2.53% and significantly enhanced callus growth, organogenesis, and cellular expansion (**p<0.01). Conversely, *Jr-miR396a* overexpression inhibited endogenous *BpGRFs*, suppressing both transformation efficiency and plant growth. These results demonstrated that endogenous miRNA-mediated regulation significantly impacts transgene performance, and that preemptive evaluation and engineering of miRNA target sites are essential for achieving stable, high-efficiency transgene expression in woody plants. The study provided a practical strategy to overcome species-specific silencing barriers in plant genetic engineering.

## Data Availability

The datasets presented in this study can be found in online repositories. The names of the repository/repositories and accession number(s) can be found below: http://xhhuanglab.cn/data/juglans.html, The Genomic Data of Juglans.
