# Supplementary material for: Escaping endogenous miRNA post-transcriptional silencing of JrGRF4b enhanced transformation efficiency in woody plants
Source: Front Plant Sci. 2025 Jun 18;16:1629166. doi: 10.3389/fpls.2025.1629166 (PMC12213585; doi:10.3389/fpls.2025.1629166)
Supplement: Supplementary file 1 [file DataSheet1.zip › Supplementary files/Supplementary Figure.pdf]

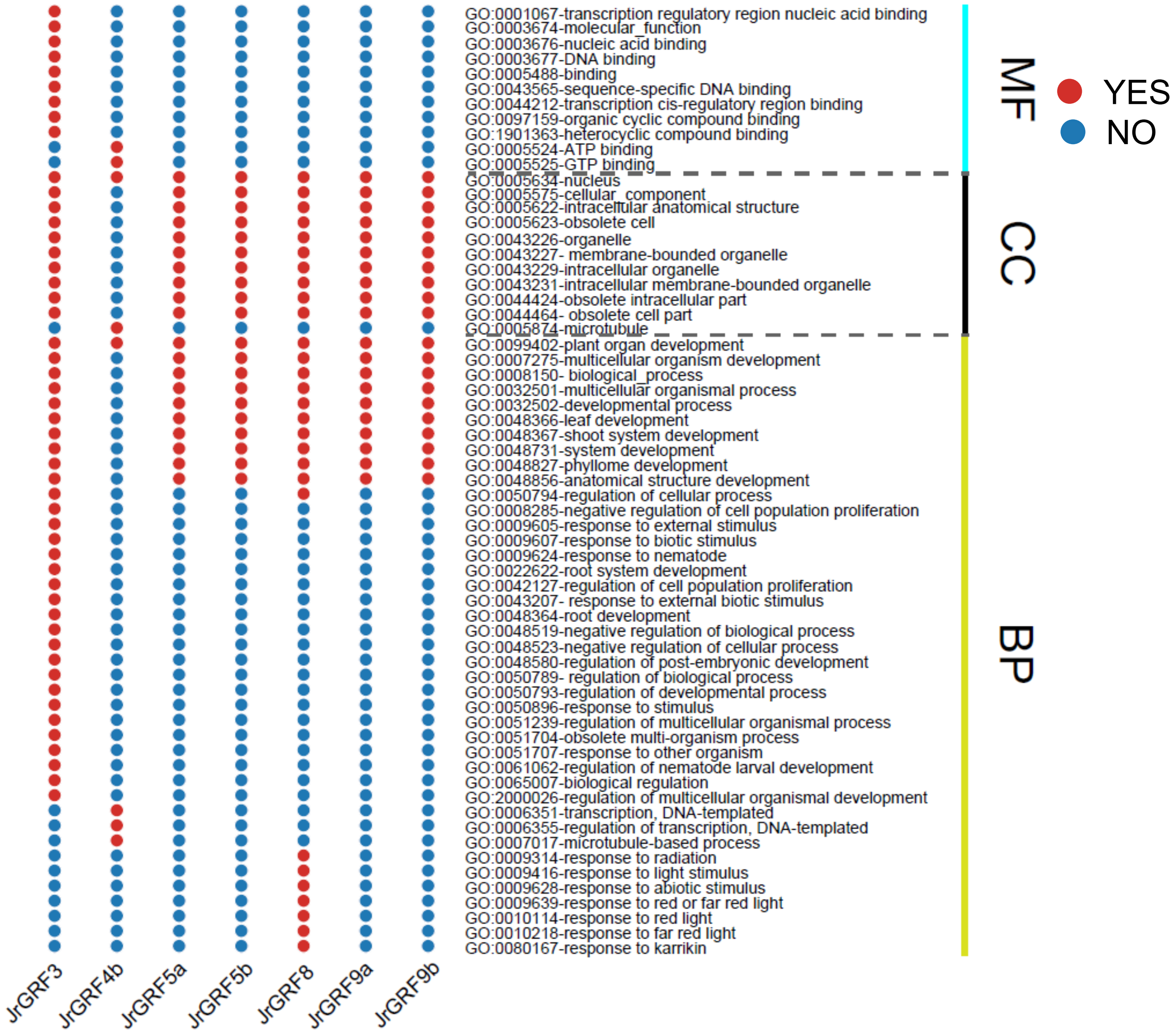

Supplementary Figure 1 Gene Ontology (GO) enrichment analysis of JrGRFs transcription factors in walnut. Red circles denoted genes enriched in the pathway, while blue circles indicate non-enriched genes. MF: molecular function; CC: cellular component; BP: biological process. In the GO annotation analysis, “YES” indicated the presence of the specified terms in the gene‘s functional classification, while “NO” denoted its absence.

Supplementary Table 1 Location and mature sequence of *Jr-miR396a* in walnut reference genome

The name of miRNAs	Location	Sequence
Jr-miR396a-1	Chr01：46080243-46080263 (+)	UUCCACGGCUUUCUUGAACUU
Jr-miR396a-2	Chr03：40486895-40486915 (+)	UUCCACGGCUUUCUUGAACUU
Jr-miR396a-3	Chr06：11494719-11494739 (+)	UUCCACGGCUUUCUUGAACUU

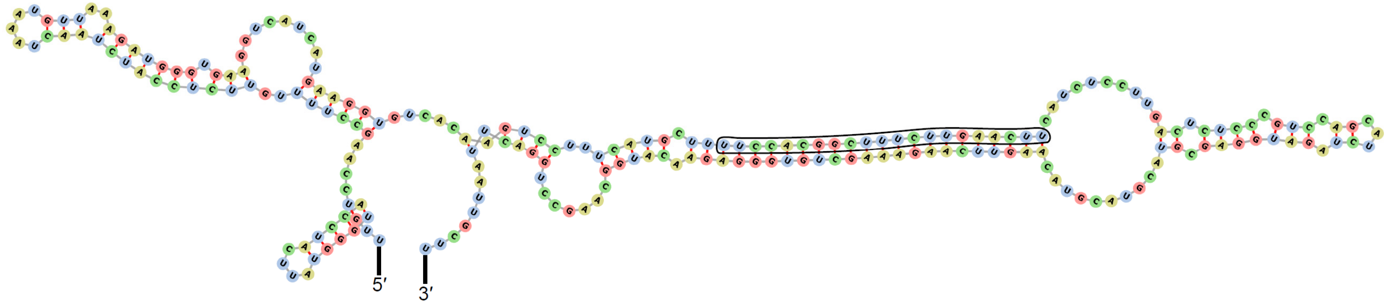

Jr-MIR396a-1

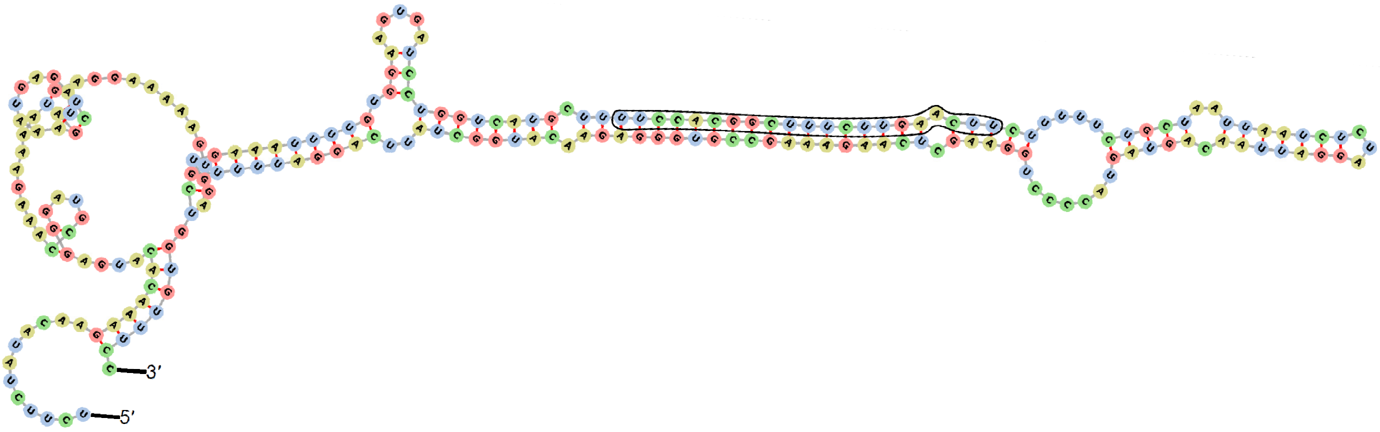

Jr-MIR396a-2

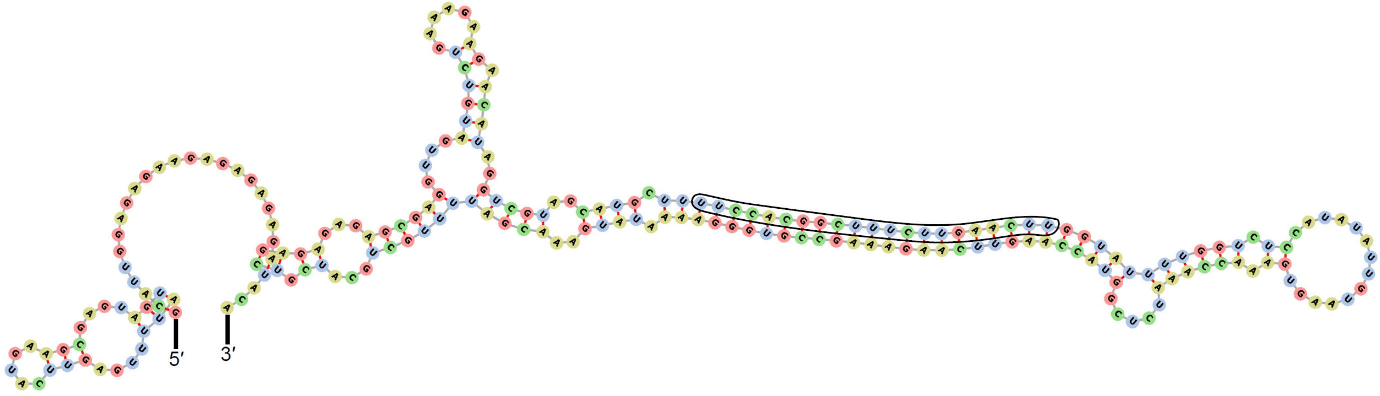

Jr-MIR396a-3

Supplementary Figure 2 Predicted secondary structures of *Jr-MIR396a* in walnut. The structure of pre-miRNAs is predicted by RNAfold (http://www.unafold.org/mfold/ applications/rna-foldingform.php). Mature miRNAs are highlighted in black frame. *Jr-MIR396* gene sequences from chromosomes 1, 3 and 6.

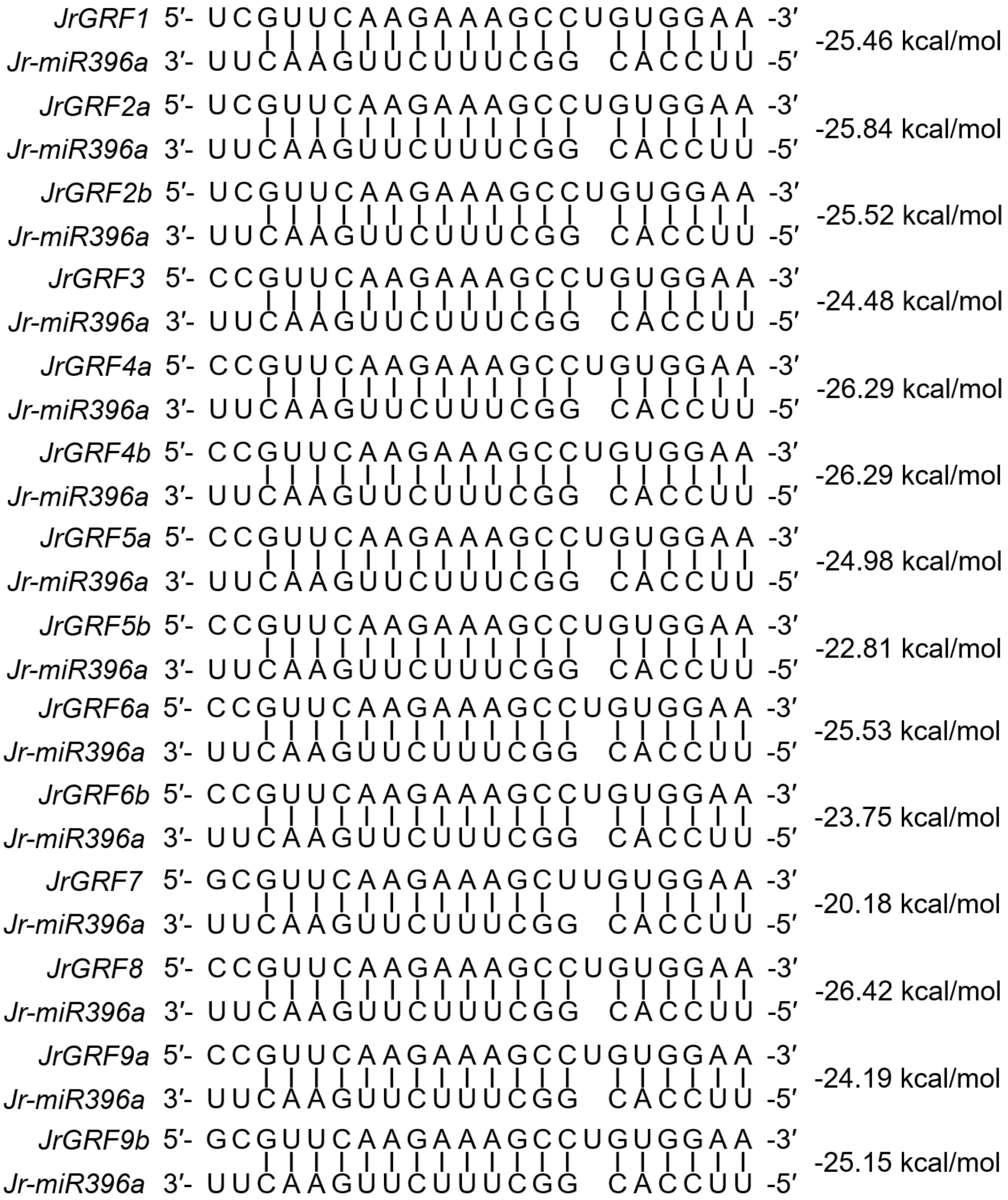

Supplementary Figure 3 Base-pairing schematic of *Jr-miR396a* and *JrGRFs* mRNA. The binding interactions between *Jr-miR396a* and the *JrGRFs* mRNA in walnut were shown. Vertical bars (I) indicate base pairing between *Jr-miR396a* and *JrGRFs* mRNA. The binding free energy (△G, in kcal/mol) for each interaction is indicated on the right.

```
JrGRF4b     5'- CCGUUCAAGAAAGCCUGUGGAA -3'
                  ||||||||||| ||||||||       -24.83 kcal/mol
Bp-miR396a  3'- UUCAAGUUCUUUC GACACCUU -5'

JrGRF4b     5'- CCGUUCAAGAAAGCCUGUGGAA -3'
                  ||||||||||| ||||||||       -24.22 kcal/mol
Bp-miR396b  3'- GUCAAGUUCUUUC GACACCUU -5'
```

Supplementary Figure 4 Base-pairing schematic of *Bp-miR396* and *JrGRF4b* mRNA. The binding interactions between *Bp-miR396* and the *JrGRFs* mRNA were shown. Vertical bars (I) indicate base pairing between *Bp-miR396* and *JrGRFs* mRNA. The binding free energy (△G, in kcal/mol) for each interaction is indicated on the right.

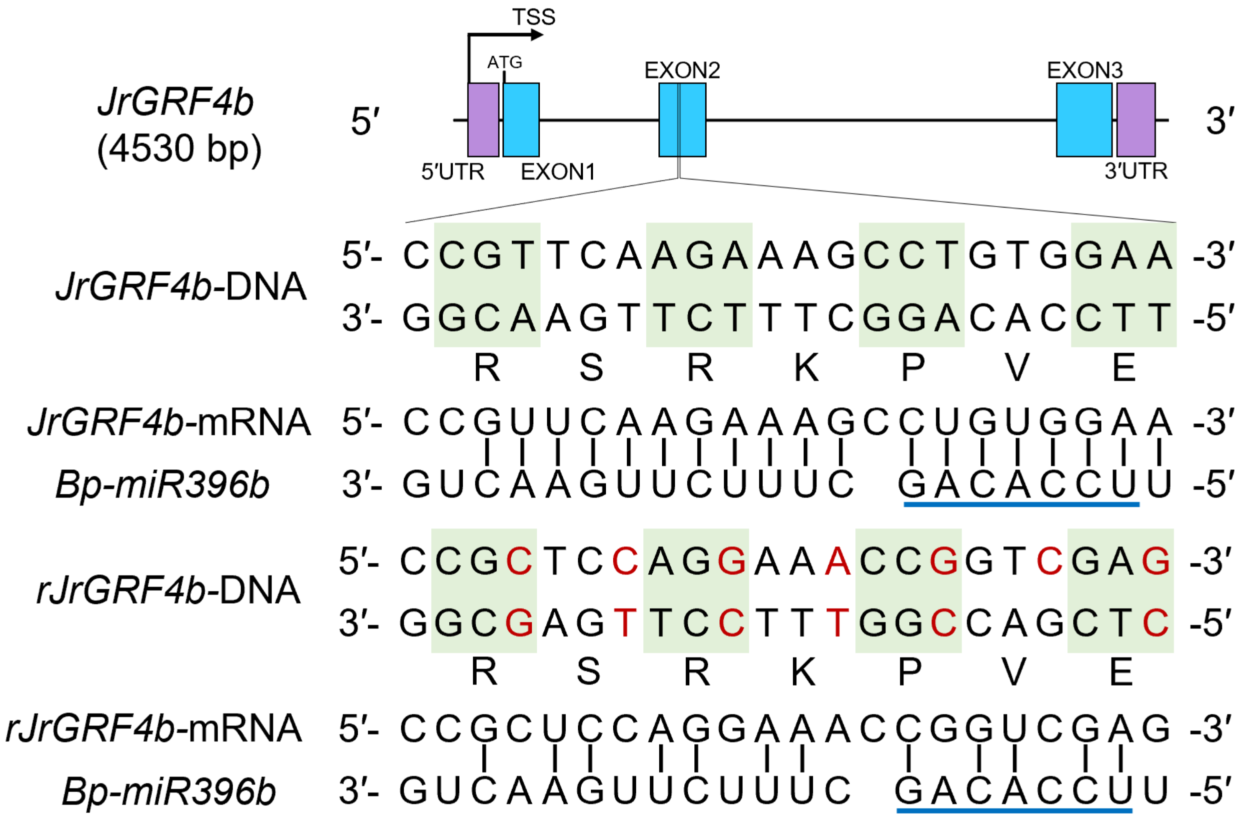

Supplementary Figure 5 Schematic representation of *JrGRF4b and rJrGRF4b* gene structure showing the *Bp-miR396b* target site. The *Bp-miR396b*-resistant *rJrGRF4b* version was introduced mutations (in red) to reduce interactions with *Bp-miR396b*. The *Bp-miR396b* seed region (nucleotides 2-8 from 5′ to 3′) was indicated by the blue highlighted line.

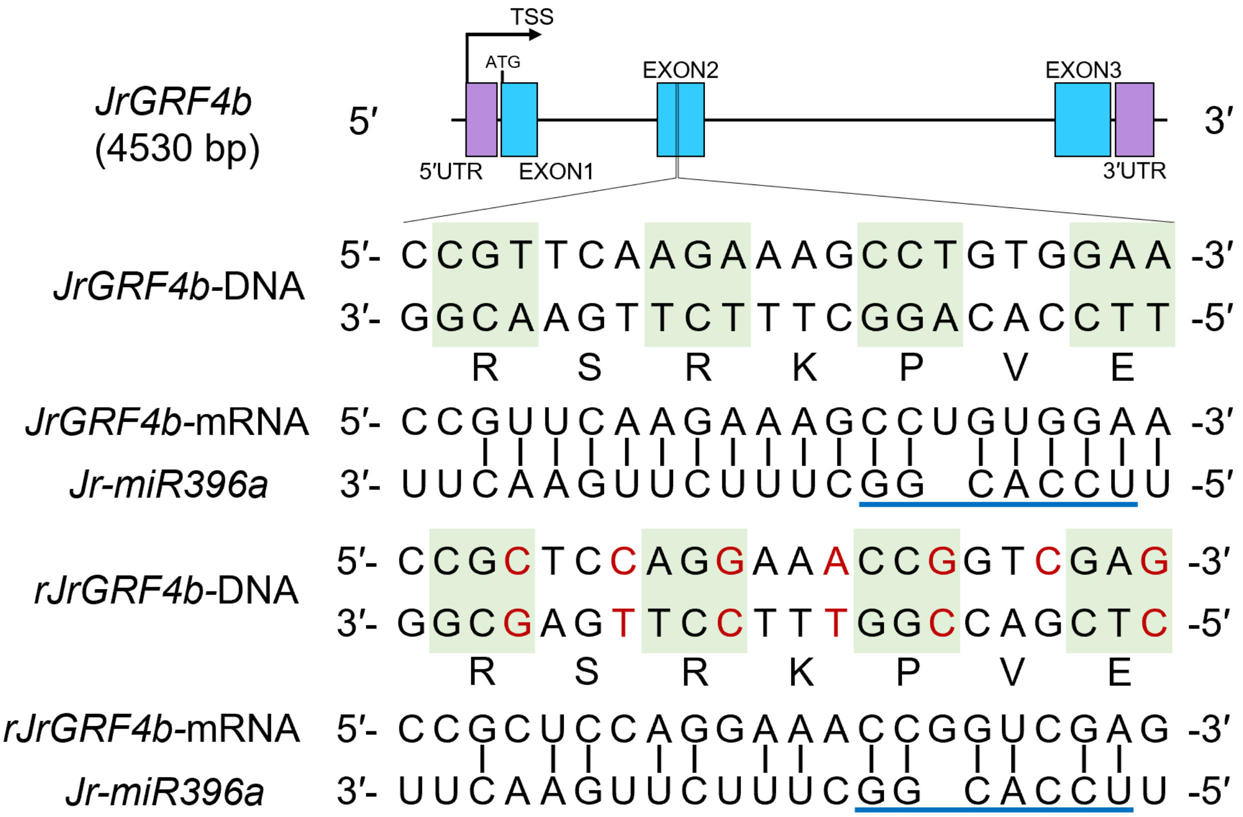

Supplementary Figure 6 Schematic representation of *JrGRF4b and rJrGRF4b* gene structure showing the *Jr-miR396a* target site. The *Jr-miR396a*-resistant *rJrGRF4b* version was introduced mutations (in red) to reduce interactions with *Jr-miR396a*. The *Jr-miR396a* seed region (nucleotides 2-8 from 5′ to 3′) was indicated by the blue highlighted line.

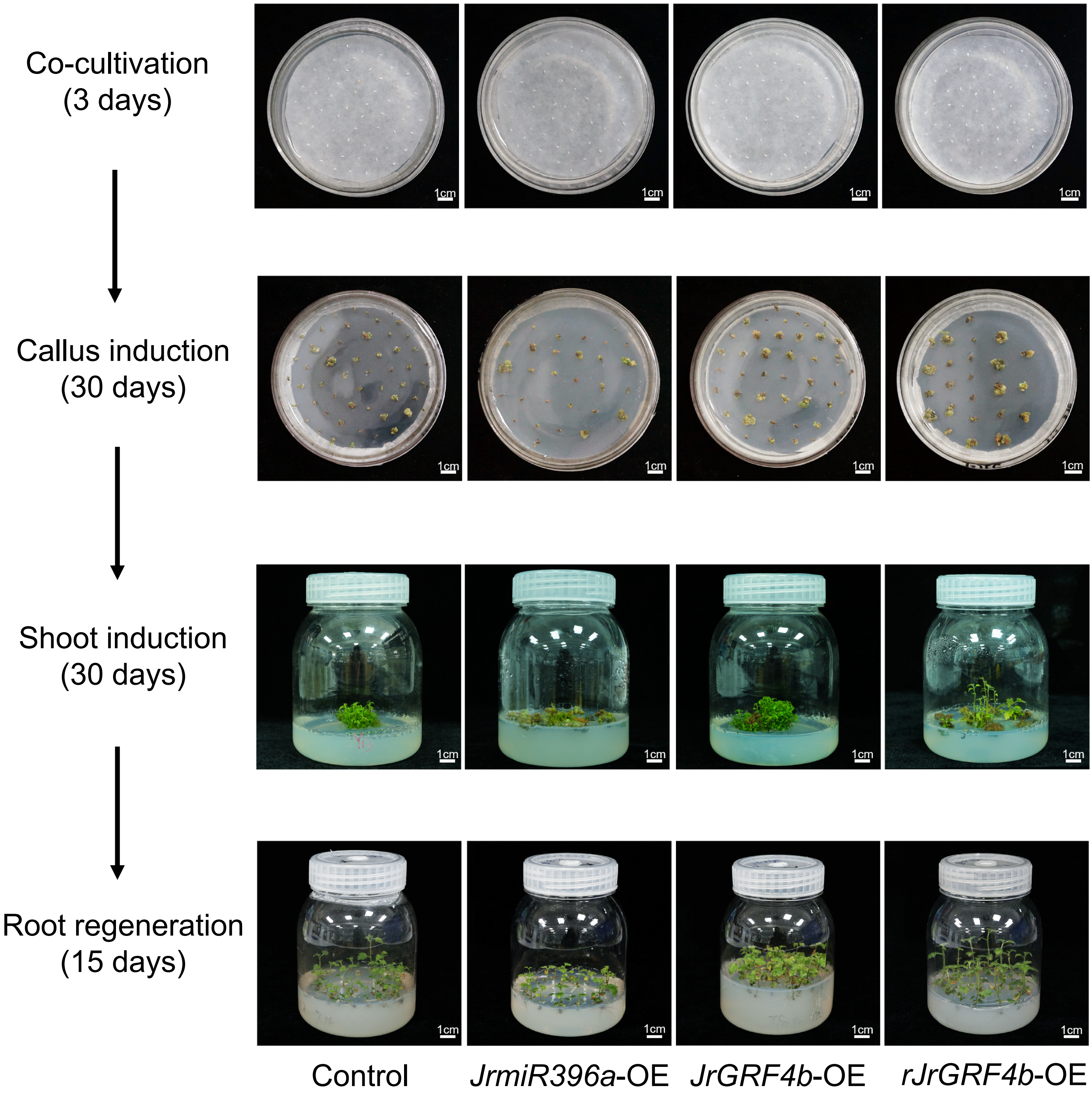

Supplementary Figure 7 Overviews of birch transformation procedures with different vector. The matured zygotic embryos from birch were co-cultivated with Agrobacterium for 3 days. Then embryos were transferred to callus induction medium for 30 days. The basta-resistant callus were transferred to shoot induction medium for another 30 days. The well-growing plantlets were transferred to rooting medium for cultivation for 15 days. Scale bar = 1 cm.

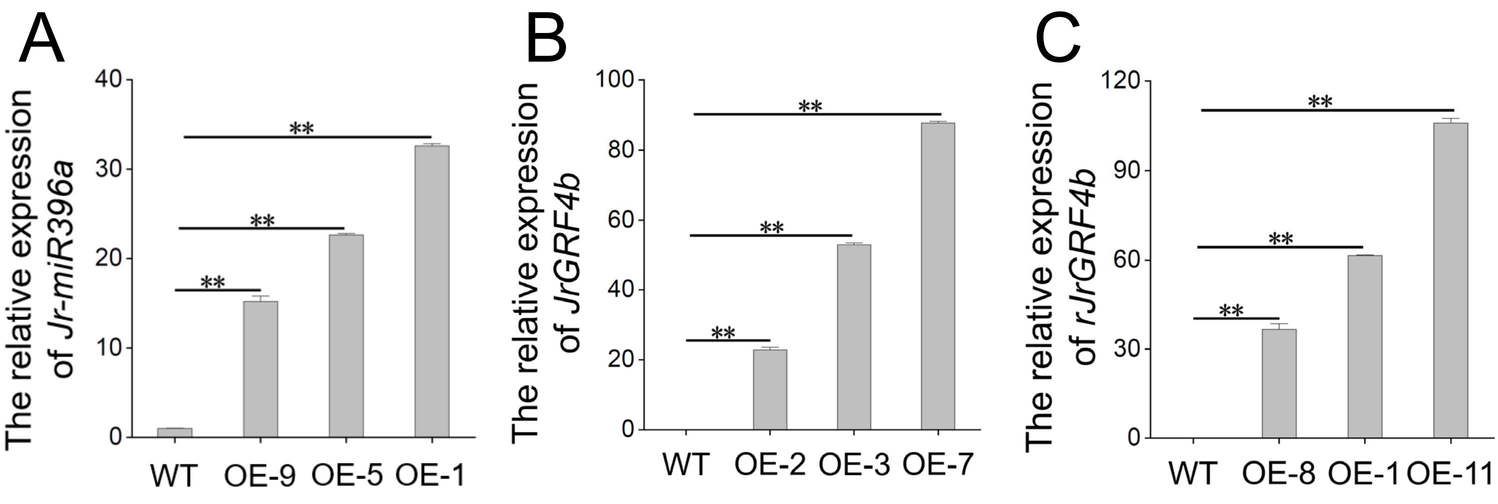

Supplementary Figure 8 qRT-PCR confirmation result of transgenic birches. (A) Analysis of the expression of *Jr-miR396a* in WT and *Jr-miR396a*-OE transgenic plants. (B) Analysis of the expression of *JrGRF4b* in WT and *JrGRF4b*-OE transgenic plants. (C) Analysis of the expression of *rJrGRF4b* in WT and *rJrGRF4b*-OE transgenic plants. *BpACTIN* was used as an internal reference gene. The error bars represent the SE of three independent biological replicates. Asterisks indicate a significant difference compared with WT plants based on Student's t-test (**$p < 0.01$).

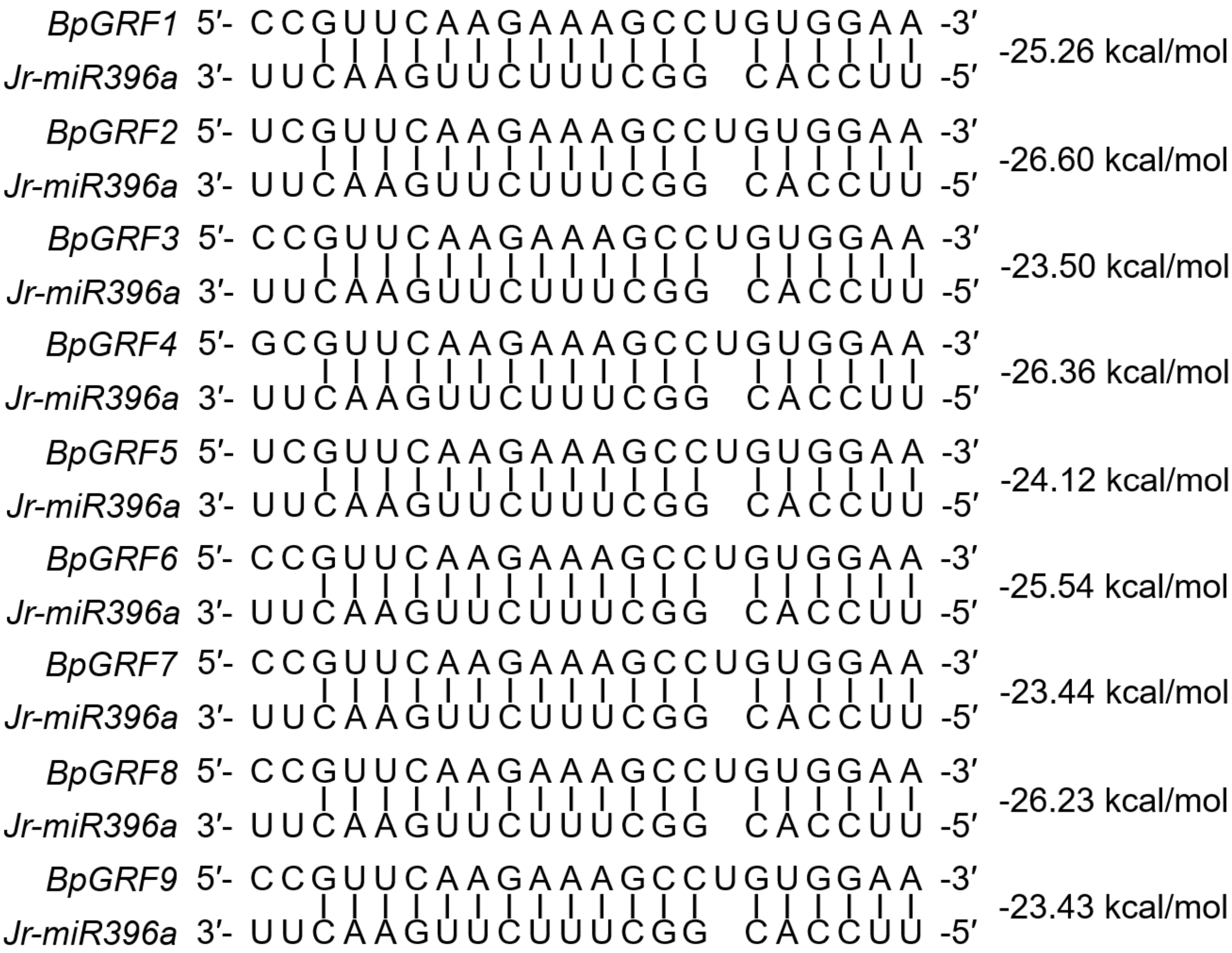

Supplementary Figure 9 Base-pairing schematic of *Jr-miR396a* and *BpGRFs* genes. The binding interactions between *Jr-miR396a* and the *BpGRFs* mRNA in walnut were shown. Vertical bars (I) indicate base pairing between *Jr-miR396a* and *BpGRFs* mRNA. The binding free energy (△G, in kcal/mol) for each interaction is indicated on the right.
